# Soft-tissue sarcomas in the head and neck: 25 years of experience

**DOI:** 10.3332/ecancer.2017.740

**Published:** 2017-06-02

**Authors:** Juan Francisco Liuzzi, Maribel Da Cunha, Daniuska Salas, Saul Siso, Esteban Garriga

**Affiliations:** 1 Head and Neck Department, Hospital Oncology Service, Venezuelan Institute of Social Security, Caracas 1040, Venezuela; 2 Head and Neck Cancer Surgery, Hospital Oncology Service, Venezuelan Institute of Social Security, Caracas 1040, Venezuela

**Keywords:** soft-tissue sarcoma, head and neck neoplasms, head, neck

## Abstract

Sarcomas are infrequent and heterogeneous tumours. They represent 1–2% of all malignant neoplasms in adults and between 4% and 10% of head and neck cancers.

**Methods:**

The research was retrospective, descriptive, and cross-sectional.

**Results:**

A study population of 62 patients with a mean age of 44 years was obtained; the most frequent location was the soft tissues of the neck (25.3%) and the mean tumour size was 7.1 cm; the most frequent diagnosis was undifferentiated pleomorphic sarcoma (25.5%) and the majority were stage III (41.4%). The lowest survival rates were associated with T2a and T2b tumours (*p* = 0.014), the presence of lymph node metastasis (*p* = 0.001), advanced stages (*p* = 0.003), and invasion of bone, blood vessels and/or nerves (*p* = 0.008).

**Conclusions:**

Late diagnosis is the main factor associated with decreased survival in patients with head and neck sarcomas.

## Introduction

Sarcomas are infrequent and heterogeneous tumours. They represent 1–2% of all adult malignant neoplasms and between 4% and 10% of head and neck cancers [1–4]. More than 50 histological varieties have been described with different biological behaviours [5]. The natural history of head and neck sarcomas is similar to that of sarcomas of the limbs; however, the anatomical complexity of this area makes surgical management difficult, since extensive surgery, with significant functional and aesthetic sequelae, is required to obtain adequate margins. Factors such as the histological subtype, degree of differentiation, and extent of the disease influence the patient’s survival and should be taken into consideration when preparing the treatment plan [5].

The purpose of this research was to evaluate the clinical characteristics and factors influencing the survival of patients with soft-tissue sarcomas in the Social Security Institute’s Hospital Oncology Service (SOH-IVSS) in the period between 1991 and 2016.

## Materials and methods

Sixty-two patients with soft-tissue sarcomas of the head and neck were treated in the SOH-IVSS between 1991 and 2016. Patients with bone sarcomas, carcinosarcomas, and Kaposi’s sarcoma, as well as patients of paediatric age, were excluded from the study.

Sarcomas were classified histologically according to the definitions set forth by the World Health Organisation (WHO), and the American Joint Committee on Cancer (AJCC) 7th edition (2010) classification was used to determine the stage of the patients’ tumours [7]. The tumour differentiation grade was established using the French FNCLCC (Fédération Nationale de Centres de Lutte Contre le Cancer [National Federation of Cancer Research Centers]) gradation system [8]. A *R*0 resection was considered when the margins were negative, *R*1 when they were microscopically positive, and *R*2 in the case of macroscopic residual disease.

The research was retrospective, descriptive, and cross sectional. The following variables were evaluated: age, sex, location, symptoms, histological subtype, tumour size, differentiation grade, the presence of nodal disease and remote metastasis, treatment performed and surgical margins.

The mean and standard deviation of the continuous variables were calculated, as well as the frequency and percentages of the nominal variables. Overall survival (OS) was analysed for the different evaluated factors. The calculation of overall survival was based on Kaplan–Meier’s non-parametric model. Survival curves were compared with the log-rank procedure and were considered to be statistically significant if *p* < 0.05. The multivariable analysis was performed using Cox regression.

## Results

The mean age of patients with soft-tissue sarcomas of the head and neck was 45 years with a standard deviation of 19 years; the time from onset of disease to the first evaluation in our hospital was 6 months: (interval 1–360); the mean tumour size was 7.1 cm with a standard deviation of 4.0 cm. There were more males than females (54.8% vs. 45.2%). The most frequent symptom was the presence of a tumour in 92%; other symptoms were dysphonia (4.8%) and nasal obstruction and/or epistaxis (3.2%). Regarding location, 25.9% originated in the soft tissue of the neck; 22.6% in the maxillary antrum; 14.5% in the oral cavity; 14.5% on the scalp; 12.9% on facial skin, among others (Table 1).

Based on the Tumour, Node, Metastasis (TNM) categorisation of tumours according to the AJCC classification, 49.9% were classified as T2b tumours, corresponding to tumours larger than 5 cm located in a deep plane. With respect to lymph node status, the most frequent category was N0 in 96.8% of cases. There was an absence of remote metastasis at the time of diagnosis in 95.2% of cases.

Regarding the clinical stage, the majority were stage III (40.2%); followed by stage Ia and Ib, with 19.4% each (Table 2).

Regarding the nuclear grade, most tumours were considered to be poorly differentiated (grade 3) at 61.3%; well-differentiated tumours (grade 1) occurred in 30.6% of cases and moderately differentiated tumours (grade 2) in 7.9% (Table 2). Undifferentiated pleomorphic sarcoma was the most frequently observed histological type (24.2%), followed by malignant peripheral nerve sheath tumours (Table 3).

Regarding the involvement of the tumour in neighbouring structures (Table 4), in 62.9% of cases, there was no evidence of involvement of the bone or neurovascular structures, whereas 30.6% had bone involvement and 6.5% involvement of blood vessels and/or nerves.

Regarding the treatment received, 43.5% were treated with surgery alone; 35.5% with surgery followed by external beam radiation therapy, and 14.3% were not able to be treated surgically. Of the patients operated on, the majority (69.8%) had wide and negative resection margins, 30.2% had microscopically positive margins and none had macroscopically positive margins.

Until the end of follow-up, 51.5% did not present with relapses, while 37.2% had some type of relapse during their treatment. It was also observed that 11.3% progressed under treatment.

**Figure 1.**
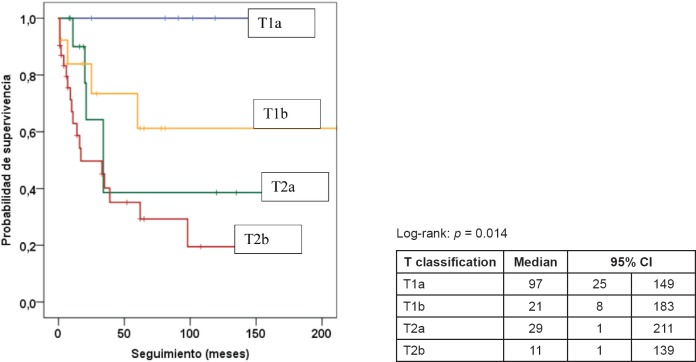
Kaplan–Meier survival curve according to T classification.

**Figure 2.**
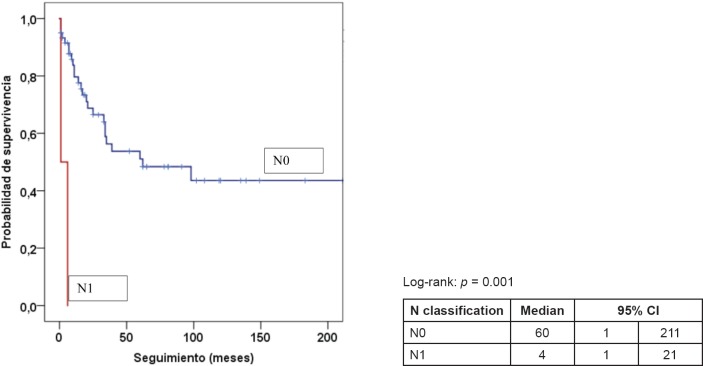
Kaplan–Meier survival curve according to N classification.

**Figure 3.**
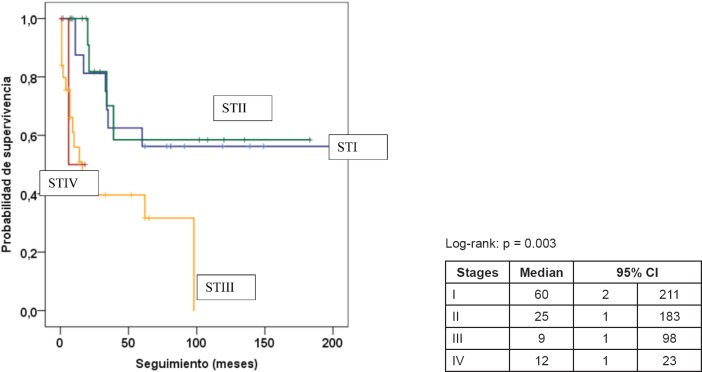
Kaplan–Meier survival curve according to stage.

**Figure 4.**
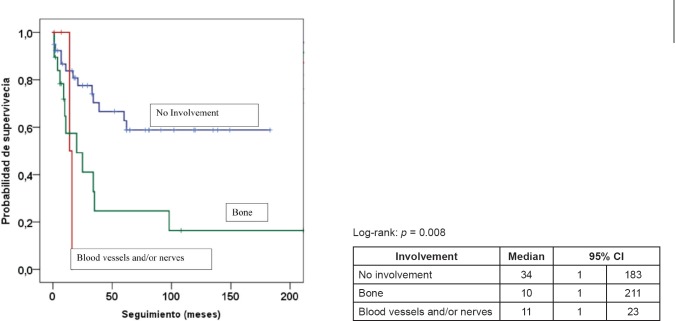
Kaplan–Meier survival curve according to involvement.

Table 5 presents the final indicators; the disease-free interval (DFI) had a median of 19 months (mean 43 months); the follow-up had a median of 20 months (mean 44 months). At the end of the follow-up, 50% of the patients were alive without disease and 35.5% dead with disease. Age, tumour size, and the presence of negative margins were not associated with survival. T2a and T2b tumours (*p* = 0.014), the presence of lymph node metastasis (*p* = 0.001), advanced stages (*p* = 0.003), and invasion of bone, blood vessels and/or nerves (*p* = 0.008) are associated with decreased survival (Figures 1–4).

T2a and T2b tumours (*p* = 0.002), stages III and IV (*p* = 0.019) and the involvement of blood vessels, nerves and/or bone were associated with decreased survival in the multivariable analysis (Table 6).

## Discussion

Head and neck sarcomas are infrequent and heterogeneous tumours. Studies of this entity are limited by the small number of patients and the different biological behaviour of the distinct histological subtypes [9].

In this research, the mean age was 45 years, the male sex was the most frequently affected (54.8%) and the mean tumour size was 7.1 cm. In general, the age of presentation is between 50 and 60 years when the paediatric population is not included [2, 9, 10]. The younger mean age of diagnosis in our study cannot be explained by early diagnosis as 70.9% of the patients had tumours larger than 5 cm.

As in most of the reports, predominance of the male sex was observed [5, 9–11]. The data on the stage of the disease at the time of diagnosis are varied, Barker *et al*. had only 11% of head and neck sarcomas diagnosed in stages III and IV [10]; in our research, 45% presented in these stages.

Differences in the way of grouping the areas where the sarcomas originate makes it difficult to compare the series. Salcedo *et al*. report in their research that the two most frequent locations were the paranasal sinuses and the soft tissues of the neck, as in our study [9]. Because of their proximity to vital structures, head and neck sarcomas present surgical difficulties and, depending on their location, may be managed with greater or lesser difficulty. Tumours located in the soft tissues of the neck or on the scalp tend to be more easily treated, but tumours located in the paranasal sinuses tend to be in the vicinity of the brain, which makes treatment more complex; this translates into a greater likelihood of positive margins after surgery.

A relevant factor in the classification of sarcomas is their histological grade; in our research, this was not associated with a decrease in survival, however, this link has been established in other research [5, 9–11].

Whenever feasible, surgery is the central element in the treatment of soft-tissue sarcomas. In our series, as in most reports, surgical treatment was performed on the majority of patients. Given the anatomical complexity of the head and neck area, obtaining negative margins is not always possible. In our research, 30.2% of the cases had microscopically positive margins; this coincides with the data reported by Salcedo *et al*.; however, in our case, this factor had no adverse effects on survival [9].

Invasion of adjacent structures is an infrequent phenomenon in head and neck sarcomas. According to Le Vay *et al*., an independent prognostic factor which is considered to be highly significant in terms of local control and survival is tumoural involvement of the bone, neurovascular structures or the skin [12]. In our study, invasion of adjacent structures was an important factor, occurring in 37.1% of cases. Invasion of neighbouring structures was a significant factor for decreased survival.

As in most series, advanced stages (III and IV), the presence of lymph node metastasis, tumour size greater than 5 cm and invasion of bone, blood vessels, and/or nerves adversely and significantly affected the survival of the patients [5, 9–14].

## Conclusions

Soft-tissue sarcomas of the head and neck are heterogeneous tumours with different biological behaviour depending on their histological type and degree of differentiation. Our research evaluated 62 patients with a diagnosis of soft-tissue sarcoma of the head and neck treated over a 25-year period. Several factors were discovered, which are considered to be prognoses of survival. One of these was tumour size; tumours larger than 5 cm usually had a worse prognosis than those measuring less than 5 cm. Another prognostic factor was the stage; patients with stage III and IV tumours had lower survival than those with earlier stage tumours. Finally, the invasion of neighbouring structures, such as bone, nerves, and/or blood vessels, which is directly correlated with increased local aggressiveness of the tumour, also represented a significant prognostic factor.

Major prognostic factors should be taken into account to determine which lesions should be considered to be potentially recurrent and to choose the most effective treatment regime.

The management of these tumours continues to be a major challenge for the multidisciplinary cancer team due to the unusual presentation, the diversity of histological types and the complexity of treatment, taking into account the proximity of the vital structures of the head and neck to the tumour.

Due to the rarity and diversity of head and neck sarcomas, it is difficult for institutions to gain much experience. To obtain better results in the study of prognostic factors, it will be necessary to combine the experience of multiple institutions in order to add a greater number of patients and to determine with greater accuracy the factors that may influence prognosis.

## Conflicts of interest.

There are no conflicts of interest.

## Figures and Tables

**Table 1.** Characteristics of the sample according to clinical and epidemiological indicators.

Variables	Statistics
Number of patients	62
Age (years) (*)	45 ± 19
Evolution time (months) (**)	6 (1–360)
Size (cm) (*)	7.1 ± 4.0
Sex	
Male	34 (54.8%)
Female	28 (45.2%)
Symptoms	
Tumour	57 (92.0%)
Dysphonia	3 (4.8%)
Nasal obstruction and/or epistaxis	2 (3.2%)
Location	
Soft tissue of the neck	16 (25.9%)
Maxillary antrum and/or nasal fossa	14 (22.6%)
Oral cavity	9 (14.5%)
Scalp	9 (14.5%)
Facial skin	8 (12.9%)
Larynx	3 (4.8%)
Oropharynx	1 (1.6%)
Ethmoidal or sphenoidal sinus	1 (1.6%)
Parotid	1 (1.6%)

(*) mean ± deviation

(**) median (minimum–maximum)

**Table 2.** Characteristics of the sample according to anatomopathological indicators.

Variables	NP %
T classification	
T1a	6 (9.7)
T1b	12 (19.4)
T2a	13 (21.0)
T2b	31 (49.9)
N classification	
N0	60 (96.8)
N1	2 (3.2)
M classification	
M0	59 (95.2)
M1	3 (4.8)
Nuclear grade	
Grade 1	19 (30.6)
Grade 2	5 (8.1)
Grade 3	38 (61.3)
Stage	
Ia	6 (9.7)
Ib	12 (19.4)
IIa	12 (19.4)
IIb	4 (6.5)
III	25 (40.2)
IV	3 (4.8)

NP = number of patients

**Table 3.** Characteristics of the sample according to the histological diagnosis.

Histological diagnosis	NP %
Undifferentiated pleomorphic sarcoma	15(24.2)
Fibrosarcoma	10(16.1)
Leiomyosarcoma	9 (14.5)
Dermatofibrosarcoma	6 (9.7)
Malignant peripheral nerve sheath tumour	5 (8.0)
Pleomorphic rhabdomyosarcoma	4 (6.5)
Angiosarcoma	4 (6.5)
Liposarcoma	4 (6.5)
Synovial sarcoma	2 (3.2)
Lone fibrous tumour	2 (3.2)
Myxofibrosarcoma	1 (1.6)

NP = number of patients

**Table 4.** Characteristics of the sample according to clinical–surgical indicators.

Variables	NP %
Involvement	
No involvement	39 (62.9)
Bone	19 (30.6)
Blood vessels and/or nerves	4 (6.5)
Treatments	
Just surgery	27 (43.5)
Surgery + POEBRT*	22 (35.5)
Surgery + POEBRT + CT**	4 (6.5)
Just CT	1 (1.6)
Just EBRT***	2 (3.2)
CT + EBRT	6 (9.7)
Margins	
*R*0	37 (69.8)
*R*1	16 (30.2)
*R*2	0
Relapse	
No relapse	32 (51.5)
Local relapse	12 (19.4)
Remote relapse	6 (9.7)
Local and remote relapse	5 (8.1)
Progression and/or persistence under treatment	7 (11.3)

NP = number of patients

* Postoperative external beam radiation therapy.

** Chemotherapy.

*** External beam radiation therapy.

**Table 5.** Characteristics of the sample according to final events.

Variables	Statistics
Disease-free interval (months)(*/**)	19/43 (1–211)
Follow-up (months)(*/**)	20/44 (1–211)
Final results	
Alive without disease	31 (50.0%)
Alive with disease	3 (4.8%)
Dead with disease	22 (35.5%)
Dead without disease	5 (8.1%)
Losses	1 (1.6)

(**) median

(*) mean (minimum–maximum)

**Table 6.** Cox regression.

Variables	RR	95% CI		p
Age ( > 45 years)	1.09	0,37	3.25	0.265
**T classification (T2a or T2b)**	**5.19**	**1.57**	**17.16**	**0.002**
N classification (N1)	1.30	0.99	2.16	0.547
M classification (M1)	1.02	0.90	2.09	0.485
Nuclear grade (G3)	0.55	0.14	1.12	0.591
**Stage (III or IV)**	**3.64**	**1.13**	**5.14**	**0.019**
**Involvement (yes)**	**2.71**	**1.06**	**6.92**	**0.037**
Margins (positive)	2.35	0.72	7.68	0.159
